# Dielectric Loss Mechanism in Electromagnetic Wave Absorbing Materials

**DOI:** 10.1002/advs.202105553

**Published:** 2022-02-07

**Authors:** Ming Qin, Limin Zhang, Hongjing Wu

**Affiliations:** ^1^ MOE Key Laboratory of Material Physics and Chemistry under Extraordinary School of Physical Science and Technology Northwestern Polytechnical University Xi'an 710072 P. R. China

**Keywords:** dielectric loss, electromagnetic wave absorption, mechanism

## Abstract

Electromagnetic (EM) wave absorbing materials play an increasingly important role in modern society for their multi‐functional in military stealth and incoming 5G smart era. Dielectric loss EM wave absorbers and underlying loss mechanism investigation are of great significance to unveil EM wave attenuation behaviors of materials and guide novel dielectric loss materials design. However, current researches focus more on materials synthesis rather than in‐depth mechanism study. Herein, comprehensive views toward dielectric loss mechanisms including interfacial polarization, dipolar polarization, conductive loss, and defect‐induced polarization are provided. Particularly, some misunderstandings and ambiguous concepts for each mechanism are highlighted. Besides, in‐depth dielectric loss study and novel dielectric loss mechanisms are emphasized. Moreover, new dielectric loss mechanism regulation strategies instead of regular components compositing are summarized to provide inspiring thoughts toward simple and effective EM wave attenuation behavior modulation.

## Graphical abstract

Electromagnetic wave loss mechanism investigation for dielectric absorbing materials is of great importance to guide the preparation of novel high‐performance absorbers. This review provides comprehensive insights into dielectric loss mechanisms and clarifies some misunderstandings and ambiguous concepts for each mechanism. In‐depth dielectric loss study and novel dielectric loss mechanisms regulation strategies are also highlighted.

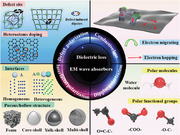

## 1 Introduction

Electromagnetic (EM) wave absorbing materials have become a research hot‐spot in recent years for their application potential in both military and civil use. As a result, stealth function for fighter can be achieved and unwanted EM wave irradiation can be prevented from threatening human health.^[^ ^1^, ^2^, ^3^, ^4^, ^5^, ^6^, ^7^, ^8^, ^9^, ^10^ ^]^ It is widely acknowledged that the requirements for excellent EM wave absorbers are well‐matched impedance and strong attenuation capacity.^[^ ^11^, ^12^, ^13^, ^14^, ^15^ ^]^ For the former, when EM wave travels from air media to absorbers’ surface, impedance match means that the impedance of absorbing materials should close to the impedance of air. As known, air media shows no EM wave attenuation ability. From this point, materials with similar insulation characteristics or low absorbing capacity will exhibit well impedance matching. However, these materials are wave transparent and EM wave can traverse them with negligible loss. While for materials with high EM wave loss ability, their impedance absolutely cannot match with the one of air. As such, it is difficult to obtain favorable impedance matching characteristics for high lossy materials. Consequently, impedance matching characteristic and strong attenuation are compromised to reach an excellent EM wave absorbing performance.

For the latter one, EM wave attenuation and corresponding mechanisms beneath impedance matching are always more attractive and gain more research attention. In general, dielectric loss and magnetic loss are the cause for EM wave dissipation.^[^ ^16^, ^17^, ^18^, ^19^, ^20^, ^21^, ^22^, ^23^, ^24^ ^]^ In comparison to magnetic loss materials, more attention is paid to dielectric loss materials investigation. Though some interesting works reported the magnetic coupling mechanism or the influence of orientation arrangement of materials under external magnetic field on EM wave loss behavior,^[^ ^25^, ^26^, ^27^ ^]^ magnetic loss materials such as magnetic metals and carbonyl iron suffer from the ferromagnetic to paramagnetic transition with temperature higher than Curie point,^[^ ^28^, ^29^, ^30^, ^31^, ^32^ ^]^ thus leading to EM loss capacity degraded. Besides, high‐density issue also restricts the development of novel magnetic loss materials. In contrast, dielectric loss will retain their loss ability even at high temperatures. In this regard, dielectric loss materials are better alternatives than that of magnetic loss materials.

Dielectric loss stems from conduction loss, interfacial polarization, defect‐induced polarization, dipole/molecular polarization, electronic polarization, ionic polarization, and atomic polarization. It is common to tailor EM wave absorbing performance through conductivity, dipoles, interfaces and defect concentration in materials to regulate conduction loss, dipole polarization, interfacial polarization, and defect induced polarization intensity.^[^ ^33^, ^34^, ^35^ ^]^ While for the other three mechanisms, ionic polarization, atomic polarization, and electronic polarization are seldom discussed. For electronic polarization and atomic polarization, they are caused by the movement of electronic cloud and atomic nucleus under external altering EM field, respectively. It is also reported that electronic polarization can be caused by hopping of electrons from Ni^2+^ to Ni^3+^ and insertion of Ni^2+^/Co^2+^ ions into the tetrahedral and octahedral positions.^[^ ^36^, ^37^ ^]^ With regard to ionic polarization, it is induced by deformation of electron cloud under EM field in ionic crystal. Unfortunately, these three mechanisms are not applicative in the commonly investigated 2–18 GHz range. Specifically, they are more profound at frequency range of 10^3^–10^6 ^GHz, which lie between infrared area and ultraviolet region.^[^ ^38^, ^39^, ^40^, ^41^, ^42^ ^]^ In other words, their contribution to EM loss at this frequency is negligible and explanation of EM wave attenuation through these mechanisms may be inappropriate. Therefore, conduction loss, dipole polarization, interfacial polarization, and defect‐induced polarization are the main contribution source to EM wave loss at gigahertz.

Recently, researches in EM wave absorption field rely on the synthesis of absorbers. It is no doubt that EM wave absorbing materials are the carriers to realize the diverse dielectric loss and subsequent EM energy dissipation, however, research attention on in‐depth mechanisms and novel models are insufficient. Numerous literatures focus on materials’ EM wave absorbing performance but explanation of EM wave dissipation is the only integration of all above mentioned four dielectric loss mechanisms. Here are two common issues for some literatures in mechanism explanation. First, some concepts about interfacial polarization, dipole polarization, and defect‐induced polarization are confused. Second, explanation of EM wave loss is empirical to some extent that is a lack of solid data support, not to mention the neglect of novel inducing mechanism in specific samples. For instance, a composite as effective EM wave absorber that contains multiple components brings about interfacial polarization but its contribution cannot be quantitative. Therefore, it is uncertain that whether promotion in EM wave loss is caused by interfacial polarization or determined by others since conductivity and defect sites are also changed in this situation. Current research model is somewhat result‐oriented. Researchers first obtain well EM wave absorbers then explain the reason for their loss of ability relying on generalizations. On the contrary, it may be better to understand the underlying relation between different mechanisms and then regulate properties of materials (including interfacial and defect engineering, dipole, and conductivity tailoring) to optimize the EM wave absorbing performance. In addition, the establishment of model to different samples is beneficial to comprehend detailed loss mechanisms. Regrettably, research on this aspect is also scarce.

**Figure 1:**
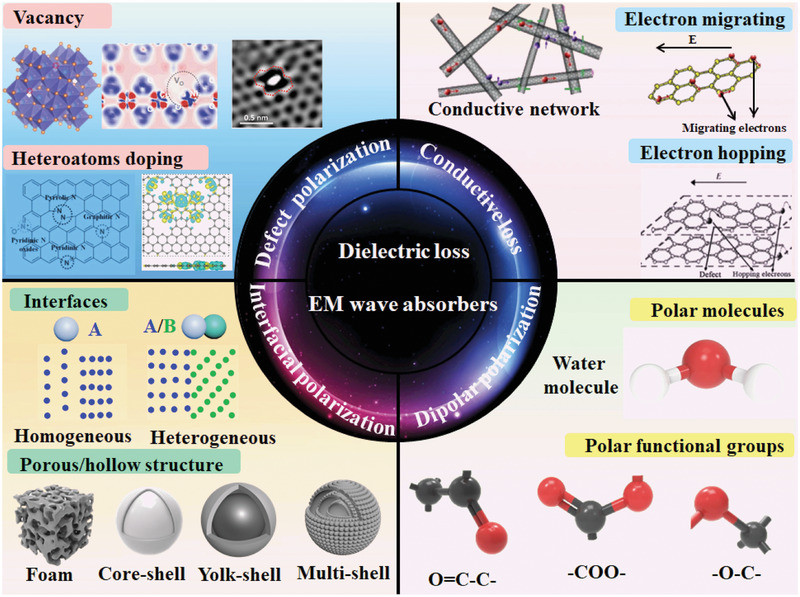
Schematic of dielectric loss mechanisms for dielectric loss materials. Top left corner is representative for defect‐induced polarization. From left to right: Oxygen vacancy in spinel ferrites. Reproduced with permission.^[^ ^109^ ^]^ Copyright 2021, Elsevier. Charge density around oxygen vacancy. Reproduced under the terms of the CC‐BY Creative Commons Attribution 4.0 International license (https://creativecommons.org/licenses/by/4.0).^[^ ^11^ ^]^ Copyright 2021, The Authors, published by Springer Nature. Defective area with atomic Ni trapped observed from TEM image. Reproduced with permission.^[^ ^67^ ^]^ Copyright 2018, Elsevier. Followed by heteroatoms doping from left to right: Schematic diagram of the bonding configurations of doping N in graphene lattice. Reproduced with permission.^[^ ^111^ ^]^ Copyright 2018, Elsevier. Charge density distributions in atom‐doping single graphitic plane. Reproduced with permission.^[^ ^128^ ^]^ Copyright 2021, Elsevier. Left bottom shows the typical interfacial polarization in homogeneous/heterogeneous interfaces and porous/hollow materials. Top right corner exhibits electron migrating and hopping model to consume EM energy and conductivity loss in conductive network. Reproduced with permission.^[^ ^132^ ^]^ Copyright 2013, Elsevier. Bottom right corner displays the dipolar polarization that is caused by polar functional groups such as ‐CO‐, ‐COO‐ and ‐C‐CO‐ and polar molecules such as water molecules.

Though plentiful excellent reviews have been reported to introduce the advances of EM wave absorbing materials,^[^ ^43^, ^44^, ^45^, ^46^, ^47^, ^48^, ^49^, ^50^, ^51^, ^52^, ^53^, ^54^, ^55^, ^56^, ^57^, ^58^, ^59^, ^60^, ^61^, ^62^, ^63^, ^64^, ^65^, ^66^ ^]^ they mainly focus on introducing EM wave loss materials on basis of material categories, such as carbon materials,^[^ ^43^, ^44^, ^45^, ^46^, ^47^, ^48^ ^]^ MOFs deprived materials,^[^ ^49^, ^50^, ^51^ ^]^ boron nitride,^[^ ^52^ ^]^ conducting polymers,^[^ ^53^, ^54^ ^]^ ferrites,^[^ ^55^, ^56^, ^57^ ^]^ sulfides,^[^ ^58^ ^]^ and MXene^[^ ^59^, ^60^, ^61^ ^]^ or design of peculiar micro/nanostructure.^[^ ^62^ ^]^ As far as we know, the topic that lies on EM wave loss mechanisms and models has been rarely reported. Herein, this review provides a comprehensive summary on dielectric mechanism study in EM wave absorption field for the purpose of supplying guidance for novel EM wave absorbers design through mechanism perspective. The conventional dielectric loss mechanisms including conduction loss, dipolar polarization, defect induced polarization, and interfacial polarization (**Figure** **1**) and corresponding misunderstandings are first summarized. Particularly, the emerging mechanisms, as well as novel model that accounts for EM wave loss, are highlighted. Finally, challenges and future research direction toward novel mechanism and models design are rationally proposed.

## 2 Dielectric Loss Mechanisms

As mentioned before, the main EM wave loss mechanisms for dielectric loss materials are interfacial polarization, dipolar polarization, defect‐induced polarization, and conductive loss. Numerous researches have explained the EM wave loss using these mechanisms and evidence has been found to prove their validity. In this part, we summarize the classification and verification techniques for each mechanism. In addition, the deficiency and misunderstanding of these mechanisms are also provided to help gain a deeper sight.

### 2.1 Interfacial Polarization

**Figure 2:**
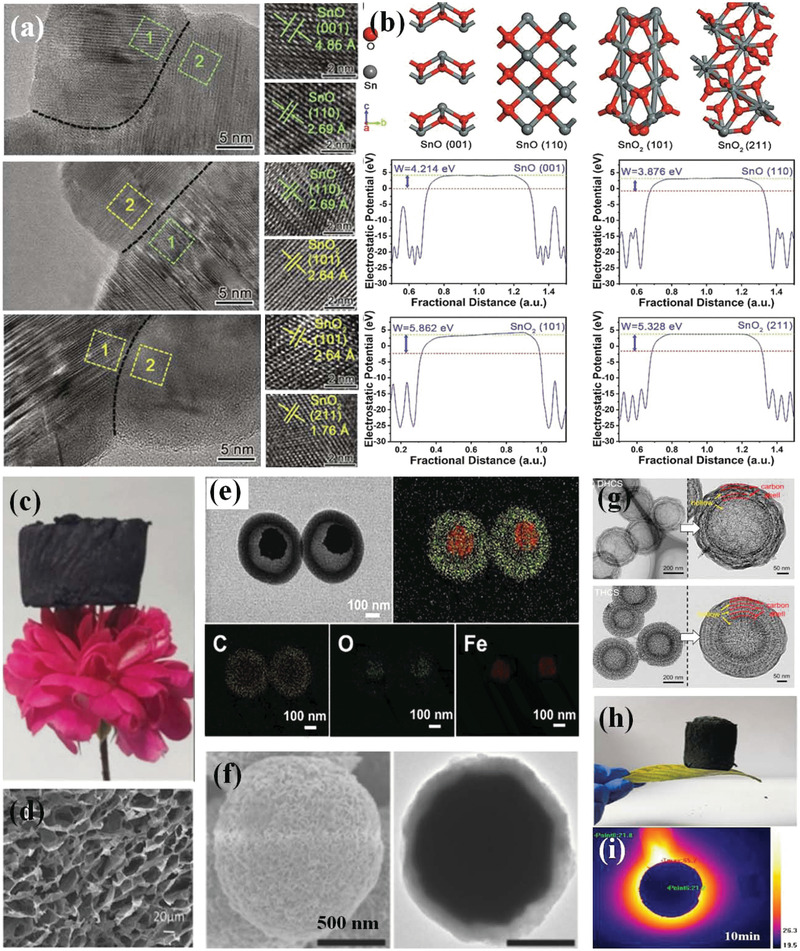
a) High‐resolution TEM images of interfaces for the SnO, SnO/SnO_2_, and SnO_2_ nanosheets. b) First‐principle calculations for the work function of the constituent planes along representative interfaces. Reproduced with permission.^[^ ^71^ ^]^ Copyright 2021, Elsevier. c) Photograph of Ti_3_C_2_T_x_ foams and d) SEM images of Ti_3_C_2_T_x_ foams. Reproduced with permission.^[^ ^87^ ^]^ Copyright 2021, Elsevier. e) TEM image and corresponding EDS mapping of yolk‐shell Fe_3_O_4_@C microspheres. Reproduced with permission.^[^ ^90^ ^]^ Copyright 2021, Elsevier. f) SEM and TEM images of CoNi@SiO_2_@TiO_2_ composite. Reproduced with permission.^[^ ^89^ ^]^ Copyright 2016, Wiley‐VCH. g) TEM images of multi‐shell hollow carbon spheres. Reproduced with permission.^[^ ^91^ ^]^ Copyright 2021, Elsevier. h) Photograph of cellulose‐chitosan framework/polyaniline hybrid aerogel and i) its thermal insulation properties. Reproduced with permission.^[^ ^88^ ^]^ Copyright 2020, Elsevier.

Interfacial polarization effect has been widely applied to enhance the dielectric loss capacity thus promoting EM wave loss. Meanwhile, interfacial polarization effect is also called Maxwell–Wagner–Sillars (MWS) effect.^[^ ^68^, ^69^, ^70^ ^]^ Up to now, two kinds of interfaces have been presented in literatures. One of which is caused by the homogeneous or heterogeneous interfaces. It is believed that differences in dielectric characteristics (ability to hold charges), as well as electrical conductivities of different components, is responsible for the redistribution of charges and subsequent interfacial polarization. Work function that represents the energetic requirements for adding or removing an electron is adopted here to explain the charge movement in EM wave absorbers. Concretely, work function of homogeneous or heterogeneous interfaces is the inducement of interfacial polarization. The varied work function would lead to the aggregation of carriers (carriers flow from lower work function to higher work function) and subsequent polarization and relaxation process under altering EM field. More precisely, interfacial polarization caused by homogeneous interface generally comes from the different work functions of lattice plane in single‐component EM wave absorbers. Lv et al.^[^ ^71^ ^]^ reported the homogeneous and heterogeneous interfaces and corresponding polarization effect in SnO and SnO_2_ samples. Both of homogeneous interfaces (SnO/SnO and SnO_2_/SnO_2_ interfaces) and heterogeneous interfaces (SnO/SnO_2_ interfaces) are present in samples (**Figure** **2**a). According to results, they discovered that compared with the heterogeneous interfaces, homogeneous interfaces were unable to provide a strong interfacial polarization effect on basis of calculated work function in Figure 2b. Specifically, work function of homogeneous interfaces for lattice of SnO and lattice SnO_2_ are 0.338 and 0.534 eV. In contrast, work function difference for SnO and lattice SnO_2_ is 1.986 eV, much higher than that of homogeneous interfaces. Therefore, heterogeneous interfaces and resultant stronger interfacial polarization are paid more attention in most cases.

The heterogeneous interfaces are readily obtained by coupling different materials. Numerous researches have been reported on compound EM wave absorbers.^[^ ^72^, ^73^, ^74^, ^75^ ^]^ Among them, composites composed of magnetic materials and high‐conductive materials are the typical representative of such absorbers. Magnetic components can provide magnetic loss while high‐conductive parts will provide dielectric loss (conductive loss), combining with interfacial polarization supplied by heterogeneous interfaces between them, strong EM wave attenuation capacity is harvest. Ferrites, magnetic metal and carbonyl iron coupled with high‐conductive carbonaceous materials (graphene, carbon nanotube) and conducting polymers are widely explored to create high‐performance absorbers.^[^ ^76^, ^77^, ^78^ ^]^ Currently, this kind of materials can be readily obtained by calcination of metal‐organic frameworks (MOFs) in inert atmosphere.^[^ ^79^, ^80^, ^81^ ^]^ Thanks to the diverse metal ions and organic ligands and well‐retained morphology of final products, researches on this aspect are popular. With regard to the evidence for presence of interfacial polarization in samples, a direct route is visualized through off‐axis electron holography technique that has been reported in many researches.^[^ ^82^, ^83^, ^84^ ^]^ Che's group designed TiO_2_@Fe_3_O_4_@PPy composite to improve interfacial polarization effect.^[^ ^83^ ^]^ According to off‐axis electron holography technique, charge density distribution around interfaces of different components was noticed. X‐ray photoelectron spectroscopy (XPS) test can be also used as indicator for interfacial polarization. Ji's group reported that coupling ferrites MFe_2_O_4_ (M = Zn, Fe, Co, Ni) with flake‐like carbonyl iron to enhance interfacial polarization.^[^ ^85^ ^]^ According to high‐resolution of Fe 2p spectra, it was found that the binding energy of Fe for the composite shift to a higher position compared to that of original carbonyl iron, which indicates the loss of electrons. Also, the value for shift may be related to interfacial polarization strength.^[^ ^86^ ^]^

The other interfacial polarization occurs on porous or hollow materials. Due to the introduction of air bubble into materials, different dielectric properties between materials and air medium may lead to the presence of interfacial polarization. Not to mention porous nature is favorable for the preparation of lightweight absorbers. However, this porous structure is more suitable for materials that possess high conductive, such as sulfide, carbon materials. Since part of materials are replaced by air medium that has no EM wave attenuation capacity, overall EM wave loss of absorbers is reduced. Therefore, high conductive materials with pore structure not only promotes impedance characteristic to some extent but also induces interfacial polarization, both of which are beneficial to elevated EM wave absorption capacity. Several porous structures including foam structure (Figure 2c,d), yolk‐shell (Figure 2e), core‐shell (Figure 2f), multi‐shelled (Figure 2g), and aerogel structure (Figure 2h) have been reported as high‐performance EM wave absorbers.^[^ ^87^, ^88^, ^89^, ^90^, ^91^ ^]^ Among which, foam structure is very attractive for its both simple preparation from templates and lightweight feature. Up to now, the foam‐structured absorbing materials including carbonaceous foam and SiC foam have been widely investigated and shown fascinating properties such as fireproofing and thermal insulation (Figure 2i).^[^ ^92^, ^93^ ^]^

According to above analysis, interfacial polarization can be promoted by two aspects. The one is to construct multiple heterogeneous interfaces by compositing diverse components while the other is to provide sufficient contact area between absorbing materials and air media in view of two kinds of interfacial polarization. Therefore, absorbing composites and porous absorbing materials fabrication are beneficial to enhance interfacial polarization loss. There are also concerns about the influence of interfacial polarization on EM wave absorbing performance. As described above, the interfacial polarization basically occurs on heterogeneous interfaces. That means multiple EM wave loss mechanisms may simultaneously exist in composite that contains diverse components. Though interfacial polarization is undoubtedly present and can be detected by off‐axis electron holography technique in different components, their contribution degree to EM wave loss cannot be quantitative. In other words, EM wave absorbing promotion may not come from interfacial polarization but originate from other mechanisms in complex materials. As for porous or hollow materials, the testing samples are obtained by coupling absorbers with adhesive such as paraffin wax and PVDF. In this situation, air bubble is filled with these adhesive. Even though the connection between materials and adhesive is physical mixture, interfacial polarization stems from heterogeneous interfaces of absorbers and adhesive. Strength of interfacial polarization is changed but it is uncertain whether the strength is enhanced or declined compared with original samples.

### 2.2 Dipolar Polarization

Dipolar or dipole polarization refers to the movement of dipoles in polar or nonpolar molecules under altering EM field. For nonpolar molecules, there is no intrinsic dipole and dipoles are generated by displacement of positive and negative charges. This process is called displacement polarization. With respect to polar molecules, rearrangement of intrinsic dipole will occur under external EM filed, thus is also known as orientation polarization.^[^ ^70^ ^]^ In this section, we focus on EM wave absorbers that contain intrinsic electric dipoles including polar water molecules and functional groups to bring about dipolar polarization. Water molecule is a typical polar molecule, which is caused by the high electronegativity of oxygen ions than that of hydrogen ions thus leading to electrons gathering around oxygen ions and subsequent uneven distribution of electron cloud in water molecule. Therefore, these polar molecules act as electric dipoles and will go through dipolar polarization and relaxation process under altering EM field, consuming EM energy. This effect can be also observed in our daily electric appliance, that is, microwave oven. The EM wave energy is transferred into thermal energy to heating food. Similarly, polar functional groups including carbonyl, hydroxide radical^[^ ^94^, ^95^, ^96^ ^]^ can also act as electric dipoles to consume EM energy due to the electronegativity difference between elements. Overall, the dipolar polarization loss in absorbers stems from the existence of polar molecules and polar functional groups.

In general, currently reported EM wave absorbers are obtained by calcination of precursors in air or insert atmosphere under high temperature. During this process, water molecules can be eliminated and polar functional groups play a more important role in dipolar polarization. Therefore, functional groups in carbonaceous materials are the main dipolar polarization source in most researches. Functional groups are rich in most carbonaceous materials including carbon fibers, carbon nanotubes, biochar, and graphene.^[^ ^97^, ^98^, ^99^, ^100^ ^]^ Besides, the emerging MXene materials also possess abundant functional groups and can provide intense dipolar polarization.^[^ ^101^, ^102^ ^]^ The presence of these functional groups can be detected by deconvolution of high‐resolution XPS spectra of C and infrared spectroscopy. C—O, C—OH, and C═O groups are common functional groups in EM wave absorbers that retains after calcination. However, the loss of oxygen‐containing functional groups occurs as the calcination temperature increased.^[^ ^103^ ^]^ Thus, elevated calcination temperature is adverse to promote dipolar polarization in absorbers for the loss of both of polar water molecules and functional groups.

Considering that dipolar polarization mainly comes from polar water molecules and functional groups, dipolar polarization loss promotion may be achieved by anchoring sufficient polar molecules and functional groups on absorbing materials. Functionalization of absorbing materials by grafting polar functional groups may be an effective strategy to enhance dipolar polarization. It has to be mentioned that in many reported literatures, authors classify defects and derived polarization process directly into dipolar polarization. These researches always refer to point defects such as oxygen vacancy and heteroatoms doping. In this situation, the electric dipoles in this condition is not intrinsic dipoles but induced by the presence of defect sites. Nevertheless, other than point defect, other defects (which will be discussed in following section) may lead to space charge accumulation and subsequent polarization instead of dipolar polarization. Therefore, simply classifying defect and induced polarization into dipolar polarization is improper. To distinguish these mechanisms, we divide these into dipolar polarization and defect‐induced polarization.

### 2.3 Defect Induced Polarization

Defect sites in absorbers can trap charge carriers and break the balance of charge distribution, thus leading to the polarization process and corresponding EM energy loss. Typically, defect sites in materials can be classified into 0D point defect (such as vacancy and dopant impurities), 1D line defect (such as edge dislocation and screw dislocation), and 2D planar defect (grain boundaries and twist boundaries).^[^ ^104^, ^105^, ^106^ ^]^ For EM wave absorbers, the common defect sites engineering is 0D point defect including oxygen vacancy regulation and heteroatoms doping.^[^ ^107^, ^108^, ^109^, ^110^, ^111^, ^112^ ^]^

**Figure 3:**
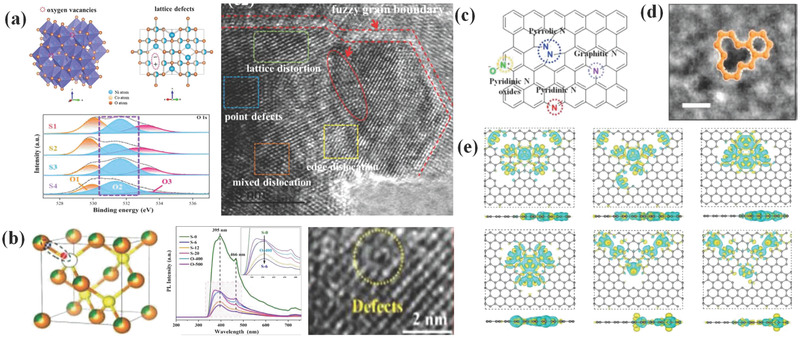
a) Schematic diagram of oxygen vacancy and crystal defect in spinel NiCo_2_O_4_ and corresponding high‐resolution XPS spectra for O 2p and TEM image. Reproduced with permission.^[^ ^109^ ^]^ Copyright 2021, Elsevier. b) Schematic diagram of defect sites in Co(OH)F/Zn_0.76_Co_0.24_S/Co_3_S_4_ composite and corresponding PL spectra and high‐resolution TEM image. Reproduced with permission.^[^ ^33^ ^]^ Copyright 2021, Elsevier. c) Schematic diagram of the bonding configurations of doping N in graphene lattice. Reproduced with permission.^[^ ^111^ ^]^ Copyright 2018, Elsevier. d) TEM image of adatom defects produced by absorbing impurity atoms. Reproduced with permission.^[^ ^110^ ^]^ Copyright 2021, Elsevier. e) Differential charge density distributions for N1, N2, B1, B2, S1, and S2 models, containing various heteroatomic bonds and defects in a single graphitic plane. Reproduced with permission.^[^ ^128^ ^]^ Copyright 2021, Elsevier.

For oxygen vacancy, it is a pervasive vacancy in oxygen‐contained materials owing to low formation energy. Due to the absence of oxygen atoms in lattice point, charge carriers generated under external alternating EM field will be trapped by oxygen vacancies, leading to the accumulation of negative carriers and following polarization. A series of characterization techniques can be adopted to detect oxygen vacancies in materials. The most common one is XPS. Oxygen vacancies and their corresponding proportion can be calculated through deconvolution of high‐resolution O 2p XPS spectra. It should be noted that XPS is a surface detection technique.^[^ ^113^ ^]^ While wavelength of EM wave at 2–18 GHz is centimeter band and can permeate absorbers. Therefore, the surface detection method XPS may not fully reveal oxygen vacancies concentration and related defect induces polarization strength. It would be more accurate to obtain oxygen vacancies concentration by combining this method with other common methods, including high‐resolution transmission electron microscopy (HRTEM), photoluminescence (PL) spectra, scanning tunneling microscope (STM), X‐ray absorption spectroscopy (XAS), positron annihilation spectroscopy (PAS), and electron paramagnetic/spin resonance (EPR/ESR),^[^ ^114^, ^115^, ^116^ ^]^ etc. **Figure** **3**a,b shows some typical techniques for testing defect sites in materials. For instance, in Figure 3a, spinel ferrite NiCo_2_O_4_ structure and its defect sites can be verified by XPS and high‐resolution TEM images. In Figure 3b, defects in samples are revealed by PL spectra and high‐resolution TEM images. Among these techniques, EPR/ESR is known to be a very sensitive method to EM wave absorption. For a high dielectric loss material, it will distort the microwave field inside an EPR/ESR resonator, which results in distortion of the signal from spins inside the material.^[^ ^117^, ^118^ ^]^ As reported in our previous research,^[^ ^119^ ^]^ EPR/ESR signals may not show oxygen vacancy but related to EM wave absorption capacity due to the work frequency of EPR/ESR being at X band, whose energy can be consumed by samples to be tested. The oxygen vacancy can be regulated by diverse methods including thermal treatment, reduction processing, and cation doping, etc.^[^ ^120^, ^121^, ^122^ ^]^ For instance, Baek et al. regulated oxygen vacancy in BaTiO_3_ by changing Ar flow rate during synthesis.^[^ ^120^ ^]^ It is found that EM wave absorbing performance of samples increases with higher oxygen vacancy concentration. Similar to oxygen vacancy, sulfur vacancy basically shares the same mechanisms as oxygen vacancy in sulfides.^[^ ^123^ ^]^

With regard to heteroatoms doping strategy, doping in carbon‐based EM wave absorbing materials is popular. In general, the perfect sp^2^ carbon lattice of carbon would be destroyed by foreign atoms doping.^[^ ^104^ ^]^ As a result, the foreign atoms with different electronegativity compared to host atoms will change the distribution of electron cloud and lead to the formation of electric dipoles in EM field, contributing to EM wave dissipation. The most common doped element in carbon‐based absorbers is nitrogen and its doping sites are shown in Figure 3c. Shao et al.^[^ ^124^ ^]^ reported an arc discharge to prepare N‐doped graphite coated cobalt nanoparticles (NPs) with different N doping contents by changing NH_3_ ratio in reaction. It turned out that samples with highest N doping content of 1.5% showed the best EM wave absorbing capacity and effective absorption bandwidth (EAB) reached to 5.6 GHz. Apart from N‐doped materials, S‐doping strategy is also effective to improve defect‐induced polarization.^[^ ^125^, ^126^ ^]^ In spite of the advance of single element doping, the influence of multiple elements co‐doping on EM wave absorption is also widely investigated.^[^ ^127^, ^128^, ^129^, ^130^ ^]^ Sun et al. fabricated N, S co‐doped reduced graphene oxide (NS‐rGO) and S, B co‐doped rGO (SB‐rGO) as EM wave absorbers. It turned out that charge density distributions are significantly affected by elements doping, which is illuminated in Figure 3e. In consequence, polarization process and resultant EM wave absorption performance are also altered.

Since defect‐induced polarization stems from the presence of defect sites on absorbing materials, defect‐induced polarization could be effectively promoted by the introduction of more defect sites on absorbing materials. As elucidated above, oxygen vacancy introduced by thermal treatment, reduction processing, and cation doping or heteroatoms such as F, N, P, and B doping strategy can elevate defect sites on absorbing materials, thus enhancing defect‐induced polarization loss. Notably, defects and induced polarization are regarded to be equal to dipolar polarization in many existing literatures. As mentioned earlier, line defects and planar defects may result in space charge accumulation and following polarization process. It is better to divide these mechanisms separately. Moreover, except for above point defects, researches on other defect‐induced polarization are scanty. Besides, grain boundary as a kind of planar defect is common in composite with multiple components. It is easy to observe the existence of defect sites on interfaces of different components for accommodating their lattice mismatch. In this regard, both interfacial polarization and defect‐induced polarization can lead to EM wave loss. However, some literatures simply classify these two mechanisms into one category, that is, interfacial polarization. In consideration of their different causes, it is more suitable to distinguish them in multiple‐components EM wave absorbers. Despite above problems, introduction of defect site in samples not only induces polarization but also alters other properties, which makes it difficult to confirm the contribution of defect‐induced polarization. Cheng et al.^[^ ^131^ ^]^ utilized sodium borohydride as reducing agent to regulate oxygen vacancy in VO_2_ samples and results showed the conductivity was also improved due to the formation of more oxygen vacancies in samples. The authors considered that EM wave absorbing capacity promotion was achieved by conjointly enhanced conductive loss and defect‐induced polarization. In other words, it is difficult to confirm the contribution of each mechanism to EM wave absorption.

### 2.4 Conductive Loss

Conductive loss or conduction loss is caused by the fact that when EM wave propagates in absorbers, their energy will be converted into electric current. During the transmission of electric current along EM wave absorbers, their instinct resistance will generate joule heat and thus consuming EM wave energy. Conductive loss exists in absorbers with high conductivity such as carbon materials (graphene, carbon fibers, and carbon nanotubes) and conducting polymers (polypyrrole and polyaniline). However, overhigh conductivity should be avoided since it undoubtedly will result in mismatch of impedance and poor EM wave dissipation capacity.

**Figure 4:**
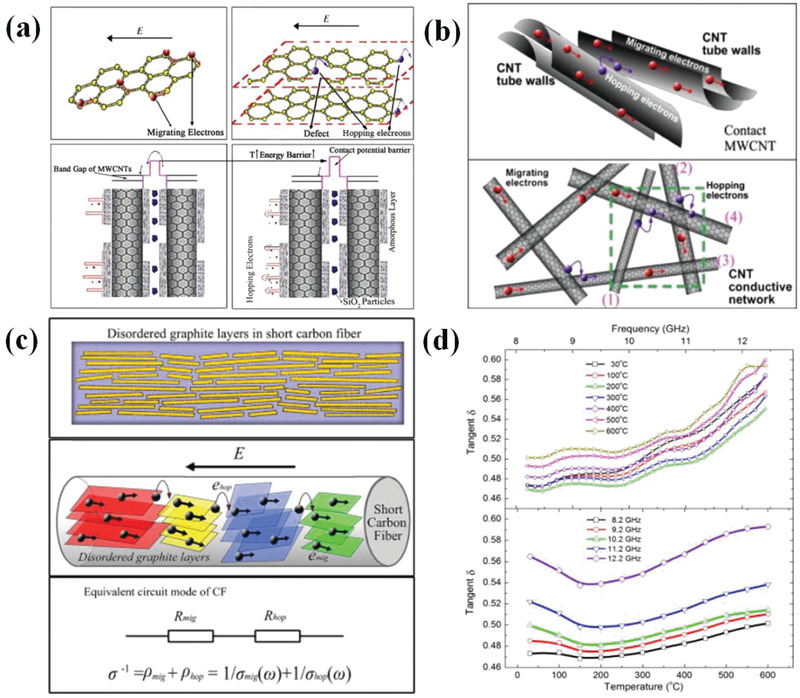
a) Electron migrating model and hopping model in CNTs/silica composites, b) electron transport in contact MWCNTs and MWCNTs network. Reproduced with permission.^[^ ^132^ ^]^ Copyright 2013, Elsevier. c) Stimulation microstructure of carbon fiber and two types of conductance occurring on the graphite layers in a CF and corresponding equivalent circuit, d) Variation trend of EM parameters for samples at different temperatures. Reproduced with permission.^[^ ^133^ ^]^ Copyright 2010, Elsevier.

Generally, two kinds of conductive loss models are established to study the conductive loss of EM wave absorbers. The first one is electron migrating model. For instance, when electric current generated under external EM field transmits in 1D carbon nanotubes or 2D graphene, it will flow along with radial direction of 1D carbon nanotubes and in plane of 2D graphene. In other words, electron migrating only refers to the free movement of electrons during propagation.^[^ ^132^ ^]^ The other one is electron hopping model that relates to electron transfer between components, interfaces, and defects. This model requires the filling of EM absorbers to be sufficient to supply conductive network.^[^ ^133^ ^]^ When filling ratio of absorbing constitution is insufficient, effective conductive network cannot be formed and the high energy barrier restricts the electron hopping process. As for the formation of conductive network condition, the energy barrier decreases to a suitable range, which will lead to hopping electrons in the network and following enhancement of micro‐current in network. In consequence, conductive loss is further strengthened in this situation. Cao's group has claimed this phenomenon in carbon‐nanotube/silica composites.^[^ ^134^ ^]^ The schematic diagram of these two kinds of models is shown in **Figure** **4**. Also, they discovered that this electron hopping effect can be promoted by elevated temperature (Figure 4d).

Conductive loss is related to conductivity of EM wave absorbing materials. As a result, conductive loss can be readily promoted by elevated conductivity of absorbing materials. Introduction of high conductive components into EM wave absorbing materials can enhance conductive loss. Meanwhile, other strategy such as increasing filling ratio can also elevate conductive loss. The measurements for conductivity of samples in literatures are direct four‐probe measurement and indirect from EIS and UV–vis DRS measurement.^[^ ^135^, ^136^, ^137^ ^]^ EIS test is widely used in electrochemistry to verify the conductivity of samples. According to the equivalent circuit and semicircle in EIS plot, a smaller semicircle means lower electrical resistivity and higher conductivity. While for UV–vis DRS measurement, the bandgap values of materials can be calculated from plots. The decline in bandgap indicates promotion in conductivity. However, in alternating EM field, variation rule for conductivity of absorbers may not behave the same as regular tests. The actual variation rule of conductivity in alternating EM fields has not been reported yet.

**Figure 5:**
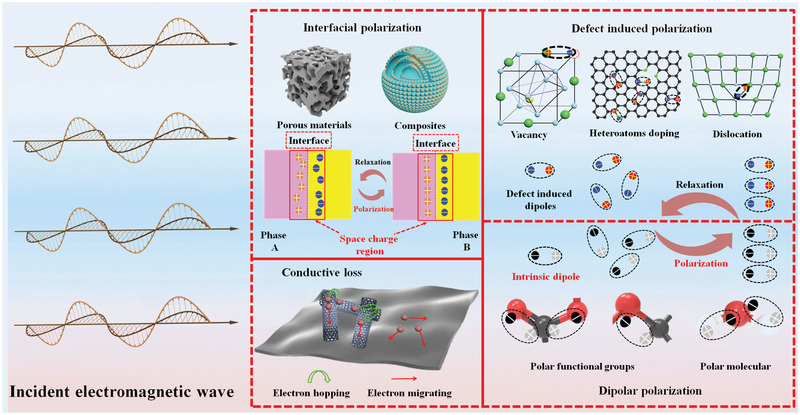
Schematic illustrations to dielectric loss mechanisms.

In this part, we discuss about the four kinds of dielectric loss mechanisms in detail. The schematic illustration for each dielectric loss mechanism is depicted in **Figure** **5**. Interfacial polarization loss can be obtained by the transition of accumulated charges on the interfaces of components with varied work functions from relaxation state to polarization state. Conductive loss is driven by the joule heat from electrons hopping and migrating under altering EM field. Among these mechanisms, dipolar polarization, and defect‐induced polarization share similarity. Some researchers consider point defect oxygen vacancy and heteroatoms doping as defect‐induced dipolar polarization or direct classify it into dipolar polarization. While for some researchers, planar defect grain boundary is regarded as interfacial polarization. For the controversial defect‐induced polarization effect, we sort out defect‐induced polarization and distinguish it from dipolar polarization and interfacial polarization, which may be helpful for mechanism analysis.

## 3 In‐Depth Mechanism Investigation

The illumination of dominated EM wave loss mechanism and novel mechanisms and models establishment is of great significance to guide the synthesis of novel EM wave absorbers. At present, it is a formidable challenge to clarify the main contribution of specific loss mechanism to EM wave loss since all dielectric loss mechanisms can provide energy dissipation but their contribution cannot be directly visualized and quantitative. Moreover, it has been discovered that novel inducing mechanisms, as well as models, are proposed in literatures to explain the new phenomenon in specific EM wave absorbing materials but they are lacking enough attention. In the following parts, efforts on main dielectric loss mechanism verification and emerging induced loss mechanism will be discussed at length.

### 3.1 Main Dielectric Loss Mechanism Investigation

The relation between different dielectric loss mechanisms is complicated. It can be described as a slight move in one part that may affect the situation as a whole. For single‐component materials, introduction of point defect not only changes the defect sites but also the conductivity. In particular, properties variation in multi‐component is more profound considering the simultaneously altered interfaces, conductivity, and defect sites (including point and planar defects). Even though, efforts on main dielectric loss mechanisms have been devoted and main contribution from EM wave absorbers is revealed in some cases.

**Figure 6:**
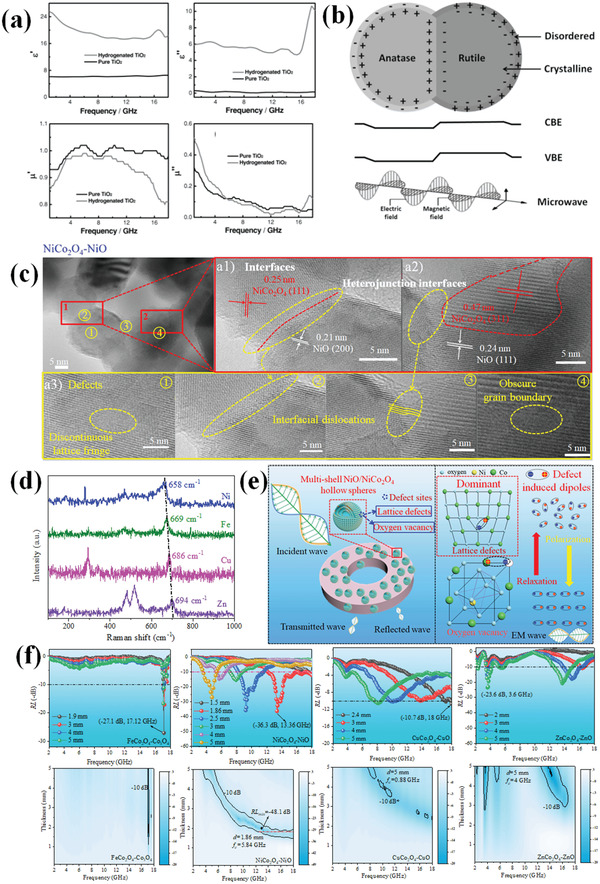
a) EM parameters of TiO_2_ samples before and after hydrogenation treatment, b) schematic diagram for interfacial polarization loss. Reproduced with permission.^[^ ^138^ ^]^ Copyright 2013, Wiley‐VCH. c) High‐resolution TEM images and abundant defect sites for multi‐shelled NiCo_2_O_4_ hollow spheres, d) Raman spectra for serial multi‐shelled MCo_2_O_4_ (M = Fe, Ni, Cu, and Zn) hollow spheres, e) EM wave loss mechanism schematic diagram for NiCo_2_O_4_ samples and f) corresponding reflection loss (RL) values for multi‐shelled MCo_2_O_4_ hollow spheres. Reproduced under the terms of the CC‐BY Creative Commons Attribution 4.0 International license.^[^ ^119^ ^]^ Copyright 2021, The Authors, published by Wiley‐VCH.

Xia et al.^[^ ^138^ ^]^ ingenious avoided complex components to construct interface but utilized a hydrogenation strategy to generate heterojunction interfaces between anatase TiO_2_ and rutile TiO_2_ phases. Hydrogenation treated sample delivered higher dielectric loss capacity, which is evident by higher complex permittivity in **Figure** **6**a. Through hydrogenation process, part of anatase TiO_2_ phase is transferred to rutile TiO_2_ phase and amorphous layer with a thickness of 2 nm was formed. To Figure out the cause of improved EM wave attenuation capacity, the authors investigated variance in conductivity, defects and polar functional groups. For defects, and polar functional groups, defects are basically unchanged according to XPS and ESR analysis while polar functional groups declined after treatment FTIR and NMR test. Therefore, other loss mechanisms can be ruled out and interfacial polarization is responsible for enhanced EM wave loss capacity. The interfacial polarization loss mechanism is schematic in Figure 6b. They also mixed anatase TiO_2_ and rutile TiO_2_ mechanically and the result turned out that this mixture is unable to provide improved EM wave absorption ability due to the absence of interfacial junctions on the nano‐meter scale.

In our previous work,^[^ ^119^ ^]^ we have successfully verified the defects (more precisely, line defect and planar defect) induced polarization was the main source for EM wave absorption in serial multi‐shelled Co‐based spinel hollow spheres. First, we selected NiCo_2_O_4_ as representative and changed their defect sites by different calcination temperatures. EM wave loss capacity was found to decrease along with increasing calcination temperature. Thanks to their identical multi‐shelled hollow spheres structure, morphology influence is ruled out in this system. With respect to other properties, an in‐depth study is carried out. Polar water molecules and functional groups are eliminated during high‐temperature calcination, thus dipolar polarization was excluded. Meanwhile, conductivity is found to decline with elevated temperature and conductive loss is also not the main contribution to EM wave loss. Similarly, more NiO generates at higher calcining temperature leading to more interfacial junctions between NiO phase and NiCo_2_O_4_ phase and thus promoting interfacial polarization. This result confirms that interfacial polarization is not the main contribution to dissipate EM energy. Only defect and its induced dipolar polarization are responsible for variance trend for serial materials. Also, abundant defect sites can be observed in TEM images in NiCo_2_O_4_ samples in Figure 6c. With respect to defect sites, oxygen vacancy and crystal defects such as planar defects exist in spinel‐structured materials. Oxygen vacancy is found to increase with higher temperatures. Therefore, it can also be inferred that oxygen vacancy is not dominated loss mechanism. After careful analysis, crystal defects display the same tendency with EM wave performance variation while oxygen vacancy shows reverse trend, revealing crystal defect‐induced polarization is the main loss mechanism. Detailed schematic diagram for defect‐induced polarization loss mechanism is present in Figure 6e. This result is also suitable for serial multi‐shelled MCo_2_O_4_ hollow sphere spinel materials. In detail, NiCo_2_O_4_ exhibits the highest defect sites in serial spinel ferrites in view of Raman spectra in Figure 6d and thus delivering the best EM wave attenuation capacity (Figure 6f).

Overall, for EM wave absorbing materials, there are always multiple loss mechanisms that coexist but contribution degrees differ. Meanwhile, their contribution cannot be quantitative intuitively, which makes it difficult to confirm the contribution degree of each mechanism. To reveal the main contribution for EM wave loss, we have to analyze the conductivity, interface and defect sites variance trends in samples seriatim according to corresponding characterization test results. Besides, systematical research should be carried out and the property variation tendency of materials should match with EM wave absorption performance trend, that make them the leading contribution to EM energy consumption. It has to be mentioned that there is a limitation for this analysis in EM wave absorption mechanisms. For instance, defect engineering in SnO_2_ materials can regulate their EM wave absorption behaviors.^[^ ^139^ ^]^ In detail, oxygen vacancy concentration was tuned by changing usage of reducing agents. During investigation, they found that conductivity increased with oxygen vacancy but the induced polarization intensity declined according to computational stimulation. However, dielectric loss capacity first increased then decreased, which exhibited a different tendency from these mechanisms. In this situation, it still cannot confirm which mechanism is responsible for the promotion of EM wave dissipation.

### 3.2 New Mechanisms/Models for EM Wave Absorption

The instinct inducement for EM wave absorption behavior variation is based on altering different dielectric loss mechanisms. Except for the mechanisms discussed above, novel mechanisms have also been proposed to reasonably explain the cause of EM wave absorption performance change in specific works.

#### 3.2.1 Interfacial Polarization Related Mechanisms

**Figure 7:**
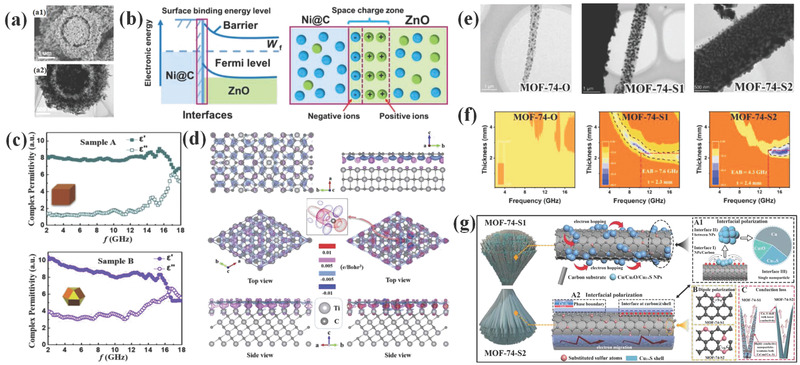
a) SEM and TEM image of yolk‐shell Ni@C@ZnO composite, b) electron transfer and induced interfacial polarization process. Reproduced with permission.^[^ ^140^ ^]^ Copyright 2020, Elsevier. c) EM parameters of samples obtained with different crystal planes, d) Charge transferring near different TiC/graphene interface structures. Reproduced with permission.^[^ ^144^ ^]^ Copyright 2020, Elsevier. e) TEM images of samples sulfuretted at different sulfur amounts, f) corresponding RL values for these EM wave absorbers, and g) schematic diagram for multiple interfacial polarization processes in S1 sample. Reproduced with permission.^[^ ^146^ ^]^ Copyright 2022, Elsevier.

Currently reported interfacial polarization is on semiconductor‐semiconductor interfaces. Another kind of interface that refers to metal‐semiconductor is called Schottky heterojunction and also designed to promote interfacial polarization.^[^ ^140^, ^141^, ^142^ ^]^ For instance, Che's group reported that yolk‐shell Ni@C@ZnO was prepared as EM wave absorber.^[^ ^140^ ^]^ SEM and TEM in **Figure** **7**a reveal its core‐shell structure. Due to the high conductivity of both metal Ni and graphitized carbon, Ni@C unit is regarded as a metalloid. Schottky contact would form at the interface of Ni@C and ZnO semiconductor, leading to the transfer of electrons from ZnO to Ni@C. Therefore, abundant electrons would accumulate at Ni@C side while schottky barrier restricts the reflux of electrons, resulting in a strong interfacial polarization effect. This process is illuminated in Figure 7b. Similar mechanism is also reported by our groups previously. Fe‐ZnO schottky contact was realized through ligand exchange method by selecting Fe(CN)_6_^3−^ to etch ZIF‐8.^[^ ^141^ ^]^ As the etching time prolonged, more Fe is formed on the ZnO surface and schottky junctions increase. Owing to strengthened interfacial polarization induced by more schottky junctions, corresponding samples deliver the best performance.

It is evident that catalytic activity of materials can be regulated by designing different exposing lattice planes due to their varied surface activity.^[^ ^143^ ^]^ As illuminated previously, the work functions of different exposing lattice planes are also variant. Accordingly, Zhang et al.^[^ ^144^ ^]^ proposed an interfacial polarization regulation strategy by altering exposed crystal facets in graphite‐coated TiC composites. Sample with TiC(100)/graphite interface and existence of TiC(100)/graphite and TiC(111)/graphite interfaces display different EM wave absorption behaviors, which is implied by EM parameters in Figure 7c. The EAB of samples with coexistence of TiC(100)/graphite and TiC(111)/graphite interfaces is 5.3 GHz, much higher than that of 1.3 GHz for TiC(100)/graphite interface sample. According to computational stimulation in Figure 7d, the surface orientation is responsible for promoted interfacial polarization and EM wave absorption ability. Notably, the orientation growth is more common in film materials due to different surface energies. But for powder materials, the crystal‐plane engineering may vastly change their morphology and other physicochemical properties.^[^ ^145^ ^]^ To sum up, crystal‐plane engineering in EM field is seldom reported to investigate interfacial polarization but exhibits the potential to tailor EM wave absorption behaviors.

Recently, our group reported an interfacial polarization regulation through particle distribution in samples.^[^ ^146^ ^]^ We attempted to tailor the particle sizes by changing amounts of sulfur powder during sulfuration of MOF‐74 precursor. A suitable sulfur addition (300 mg, S1) leads to uniformly multiple NPs supported on carbon substrate while excessive sulfur addition (600 mg, S2) results in the coverage of sulfide layer on the surface of carbon substrate. The morphologies of samples are shown in Figure 7e. As a result, S1 sample displayed the best EM wave absorption performance with EAB of 7.3 GHz (Figure 7f). Through in‐depth study, it is found that the conductivity and defect‐induced polarization are comparable. Thus, it is believed that interfacial polarization is accounted for promoted dielectric loss capacity. There are three kinds of interfacial polarization sources in S1, including interfacial polarization from single NPs that contain multiple components, NPs and carbon substrate, and interfaces between different NPs, which is schematic in Figure 7g. With regard to S2, it lacks such interfacial polarization mechanisms due to the formation of sulfide layer carbon substrate surface, thus exhibiting much worse EM wave absorbing performance.

#### 3.2.2 Defect Induced Polarization Related Mechanisms

In normal spinel structure AB_2_O_4_, metal A occupies the centers of locating at tetrahedral sites, metal B occupies octahedral sites and O^2−^ sits at the polyhedral vertexes. Interestingly, relative occupancy of divalent and trivalent cations in the octahedral and tetrahedral sites can be readily tuned in spinel structure. As a result, it is easy to introduce defect sites in spinel structure, thus tuning the properties of spinel ferrites to regulate their EM wave dissipation performance.^[^ ^147^ ^]^

Our group discovered novel EM wave absorption mechanisms in spinel ferrites by regulating the valence state of ions and ionic mobility in spinel crystal.^[^ ^148^, ^149^ ^]^ For regulation valence state of ions mechanism, three kinds of NiCo_2_O_4_‐CoNiO_2_ composites were prepared at different calcination temperatures.^[^ ^148^ ^]^ Uncalcined NiCo_2_O_4_‐CoNiO_2_ sample displays amorphous nature with a low Co^2+^/Co^3+^ ratio of 0.59. Along with increment in annealing temperature from 450 to 650 ℃, the diffraction peaks appear and ratio between Co^2+^ and Co^3+^ first increases to 1.76 and then declines to 1.20. The ascending Co^2+^ is caused by the reduction of carbon during calcination while higher temperature of 650 ℃ would lead to the oxidation of Co^2+^ again. During this valence state transfer process, disorder in lattice is induced and abundant oxygen vacancy sites are generated. As a result, EM wave absorption capacity could be enhanced. Sample with highest Co^2+^/Co^3+^ ratio of 1.76 that shows the best EM wave absorption performance verifies this conclusion.

With respect to ionic mobility mechanism, Co_1.29_Ni_1.71_O_4_ hollowed‐out spheres ferrites were synthesized at different calcination temperatures to reveal this mechanism.^[^ ^149^ ^]^ Samples harvested at different temperatures share the same XRD patterns but they differ in ion distribution in spinel crystal. Generally, ions in this spinel could be expressed as Co_a_^2+^[Co_1.29‐a_^3+^Ni_1.71+a_^3+^]Ni_1‐a_^2+^O_4_, in which trivalent cations in square bracket locate at octahedral sites while the other divalent cations locate at tetrahedral sites. We discover that ionic mobility occurred with increment of annealing temperature. According to XPS analysis, Co^2+^ and Ni^3+^ ratio increase while Co^3+^ and Ni^2+^ ratio decrease along with higher temperature. Based on the above formula, Co^3+^ is reduced to Co^2+^ and enters into tetrahedral sites while Ni^2+^ is reversely oxidized to Ni^3+^ and enters into octahedral sites. In other words, calcination induces the position exchange of nickel ions and cobalt ions in spinel structure. This process accompanied with abundant defect sites and lattice disorders would result in the generation of abundant oxygen vacancies and following favorable EM wave attenuation ability. Among these samples, the one calcined at 550 °C exhibits the widest EAB of 5.13 GHz, which is brought about by the highest oxygen vacancy due to strongest ionic migrations at this temperature.

#### 3.2.3 Macroscopical Morphology Related Mechanisms

Commonly, multiple reflections are reported in materials that are related to UV and visible region applications. Wavelengths of them are nanoscale that are comparable to the size of micro/nanomaterials, thus multiple reflections process is tenable. EM wave in microwave band, however, possess centimeter wavelength and can traverse absorbers in micro/nano scale. Therefore, micro/nano morphology may be more profound in altering materials’ physicochemical properties to change EM wave absorption ability. In comparison, macroscopical morphology regulation is more visualized to investigate its influence on EM wave attenuation. Here, several typical works are collected to illuminate the influence of macroscopical morphology on determination of EM wave absorption behaviors.

Ji's groups revealed the connection between microscopic dielectric loss and macroscopical morphology effect on EM wave dissipation of absorbers’ by utilizing 3D print technique.^[^ ^150^ ^]^ They first studied the EM wave absorption behavior of CNTs. Then numerical simulation was employed to optimize 3D periodic structures of EM wave absorbers. With optimized parameters of height and tip configuration, stimulated structure displayed a superior performance. Thereafter, above CNTs were mixed with suitable resin as print ink to prepare EM wave absorbers with periodic structures by 3D print to deliver excellent absorbing performance. Other than this work, morphology design with diverse inks is also realized.^[^ ^151^, ^152^ ^]^

**Figure 8:**
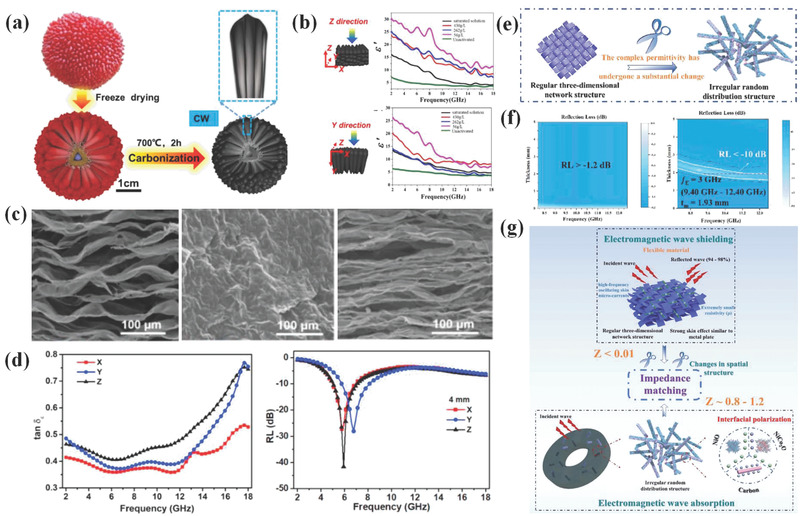
a,b) Schematic diagram for synthesis of waxberry‐based EM wave absorbers and their diverse EM parameters along different directions. Reproduced with permission.^[^ ^154^ ^]^ Copyright 2021, Elsevier. c) SEM images of MXene aerogels at different directions, d) corresponding dielectric loss tangent values and RL values. Reproduced with permission.^[^ ^157^ ^]^ Copyright 2020, Elsevier. e) Scissoring treatment for flexible carbon cloth‐based EM wave absorbers, f) RL values curves for samples before and after scissoring treatment in X band, g) Schematic diagram of EMW shielding and absorption mechanism. Reproduced with permission.^[^ ^159^ ^]^ Copyright 2021, Elsevier.

Che's group took advantage of natural hierarchical gradient structure of waxberry as EM wave absorbers to tailor EM parameters and attenuation capacity.^[^ ^153^ ^]^ After carbonization process, sample rings prepared with the morning glory‐like outer layer were sensitive to states and arrangement mode. When being arranged along radial (Z‐axis) and axial (Y‐axis) directions, samples showed different EM parameters. The hierarchical structure of waxberry was responsible for anisotropic parameters. Similar work is reported by Sun et al.^[^ ^154^ ^]^ using the same materials to construct EM wave absorbers (**Figure** **8**a). The variances of EM parameters along different directions are shown in Figure 8b. Other than this material, MXene‐based and wood‐based EM wave absorbers also behave anisotropic EM parameters due to their morphology.^[^ ^155^, ^156^, ^157^, ^158^ ^]^ For instance, not only the morphologies of samples are varied from different directions (Figure 8c) but also the EM parameters and loss capacities differ (Figure 8d). Further, when ball‐milled treatment was applied to outer layer in Che's work, EM parameters of corresponding sample were dramatically decreased. This phenomenon could be ascribed to the radically destroyed morphology and altered properties of sample.

From the above work, we can be aware that it is inappropriate to describe the microscopic loss mechanism combined with initial morphology and properties, especially for flexible EM wave absorbing materials. Even though grinding process may only refer to physical changes, their structure is destroyed and it may also alter the EM wave propagation characteristic in absorbers thus leading to varied even reversed EM wave absorption behavior. Inspired by this work, our group focused on the flexible EM wave absorbers and investigated their shielding and absorption transfer characteristics through morphology evolution (shown in Figure 8e).^[^ ^159^ ^]^ In detail, NiCo_2_O_4_/NiO composites were generated on carbon cloth and as‐prepared samples were calcined to form flexible hybrids. When test EM parameters of samples by waveguide method at X band, the samples possess high conductivity and behave like metal plates that showed strong shielding effect due to skin effect, which are visual by RL values in Figure 8f. Therefore, 3D connected network structure of hybrids is destroyed by cutting in into randomly distributed structures with scissor. This sample is tested again in coaxial method at the same X band and exhibit EAB of 3 GHz (Figure 8f). Through this morphology evolution, flexible EM wave absorbers will go through from shielding to absorption function transition due to improved impedance match characteristic (Figure 8g).

#### 3.2.4 Other Mechanisms

**Figure 9:**
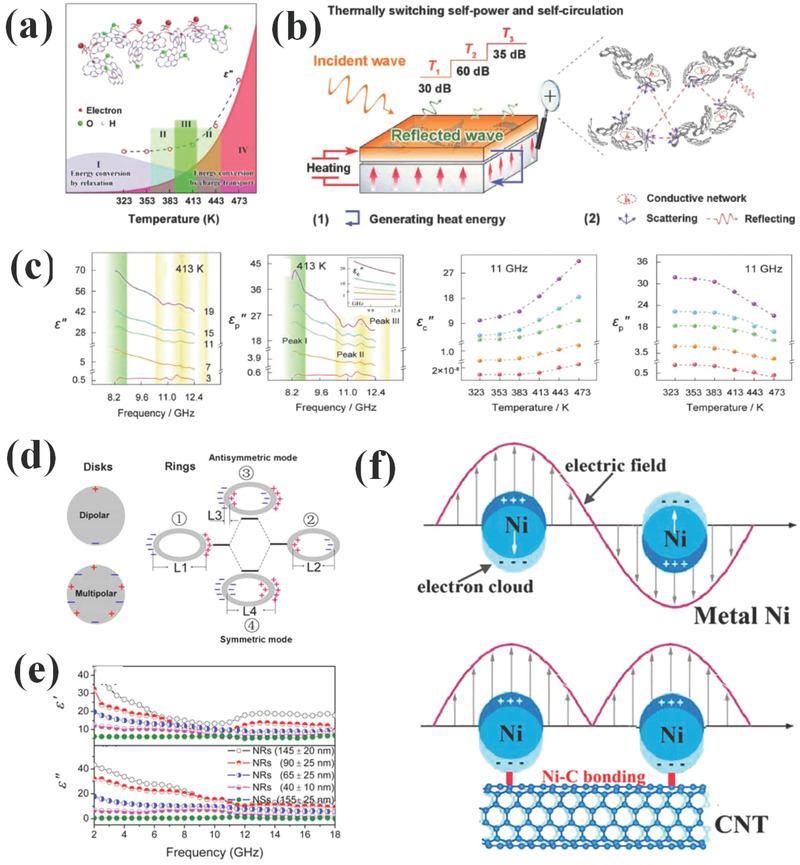
a) Relationship of relaxation contributed *ε*_p_″ and charge transport contributed *ε*_c_″ versus temperature, b) Schematic illustration for energy conversion of graphene‐SiO_2_ with self‐powered and self‐circulated system, c) EM parameters of different graphene content and temperature characteristics of *ε*_c_″ and *ε*_p_″ at *f* = 11 GHz. Reproduced with permission.^[^ ^160^ ^]^ Copyright 2018, Wiley‐VCH. d) Polarization and coupling modes in nanorings and nanospheres and e) Frequency dependence of the real and imaginary part of complex permittivity Fe_3_O_4_ nanorings at different sizes. Reproduced with permission.^[^ ^161^ ^]^ Copyright 2016, AIP Publishing. f) Schematic of surface electric fields of metal nickel and Ni—C bonded Ni‐CNT composite. Reproduced with permission.^[^ ^165^ ^]^ Copyright 2017, American Chemical Society.

A competitive synergy mechanism was reported by Cao's group.^[^ ^160^ ^]^ Graphene‐SiO_2_ composite was prepared to illuminate this mechanism. It was found out that both polarization relaxation and conductive loss were contributed to EM energy loss in graphene‐SiO_2_ composite while their contribution differed at different temperatures. Polarization relaxation was dominated at lower temperatures but would fade at higher temperatures due to depolarization of dipoles. On the contrary, conductive loss was more profound at higher temperatures owing to promoted electrons migration and hopping process. By tuning temperature, EM wave loss would gradually change from polarization relaxation dominated section (I) to conductive loss section (IV), which is shown in **Figure** **9**a. During EM wave absorption, heat is generated and spread due to well thermal conductivity of graphene, leading to the promotion of conductive loss as well as EM dissipation (Figure 9b). This result is proved by the elevation of conductivity loss portion in complex permittivity, which can be observed in Figure 9c. They also succeed in extending this thermal‐driven strategy in graphene‐Fe_3_O_4_ composite.^[^ ^110^ ^]^

Another mechanism, namely, plasmon resonance has also been adopted to explain EM wave loss promotion in absorbers.^[^ ^161^, ^162^, ^163^, ^164^, ^165^ ^]^ For example, Tong et al. prepared elliptical Fe_3_O_4_ nanorings as EM wave absorbers and discovered that electron oscillation paths varied due to the unique anisotropic morphology (Figure 9d).^[^ ^161^ ^]^ Besides, EM parameters are also found to change with sizes of nanorings (Figure 9e). They further extend this mechanism into Cu/C and Co/C/Fe/C composites.^[^ ^162^, ^163^, ^164^ ^]^ Similarly, Sha et al. reported plasmon resonance mechanism in Ni‐CNTs composite by generating Ni—C bond between these two components thus altering surface distribution of electric field and the loss mechanism is present in Figure 9f. Notably, plasmon resonance is common in visible region or near‐infrared region. There are literatures that take advantage of this mechanism to explain EM wave absorption behavior in metamaterials at terahertz.^[^ ^166^ ^]^ Nevertheless, corresponding data support of this mechanism in microwave frequency may need to be further validated.

## 4 Novel Strategy for Dielectric Loss Mechanism Regulation

It has been acknowledged that EM wave absorbers with single component cannot fulfill impedance match characteristics and loss capacity simultaneously. Therefore, current solution is to couple different components into composites as EM wave absorbers with multiple loss mechanisms. However, this compositing process often refers to complex procedures and strict reaction condition requirements, which is absolutely time‐consuming and adverse to scalable production. To overcome these drawbacks, novel strategies are proposed for simplified synthesis and achieving multiple loss mechanisms manipulation. Besides, novel manipulation strategies other than regular composition adjustment are also proposed to effectively tune EM loss mechanisms and will be discussed below.

### 4.1 Novel Synthetic Strategy

Conventional routes for EM loss mechanism are basically relied on coupling of high‐conductive materials with lower ones to eliminate skin effect or dielectric‐loss materials with magnetic‐loss materials to achieve synergetic EM wave absorption. This compositing process means multiple synthetic procedures. To avoid complicated reaction process, significant efforts have been devoted to simplify materials synthesis but maintaining effective EM loss mechanism regulation. In this part, several typical cases for common metal sulfides and metal/metal oxides materials are summarized to clarify current efforts on EM wave loss mechanism regulation regardless of complex constitution coupling.

#### 4.1.1 Metal Sulfides

Metal sulfides as EM wave absorbers have become a research hotspot due to their high conductivity and strong dielectric loss capacity. However, they alone lack magnetic loss part and show unsatisfied absorption performance due to mismatch of impedance. Though there are numerous researchers tried to optimize the absorption performance of sulfides via combining them with carbon materials, metal NPs or other sulfides,^[^ ^167^, ^168^, ^169^ ^]^ this component introduction is often performed blindly and based on semiempirical rules, lacking precise modulation of components, interfaces, and defects during the reaction process.

**Figure 10:**
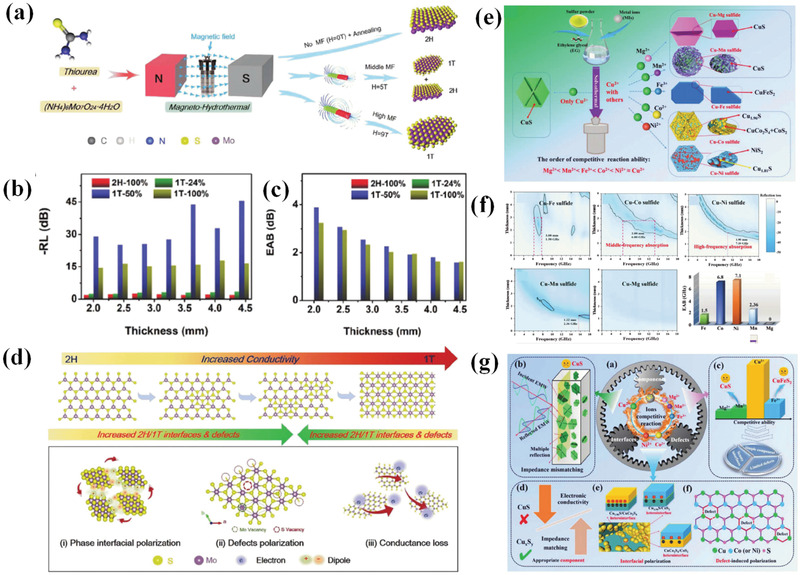
a) Schematic illustration of the synthesis route of MoS_2_ with pure or mixed phases, b) RL and c) EAB comparison curves of absorbers under the same thicknesses, d) Phase‐dependent conductivity, 2H/1T interfaces, and defects of MoS_2_ and corresponding loss mechanisms. Reproduced with permission.^[^ ^170^ ^]^ Copyright 2021, Wiley‐VCH. e) Schematic of synthesis process for serial sulfides, f) 2D RL plots of EAB for serial sulfides, g) Schematic diagrams of EM wave absorption mechanisms for Cu‐M sulfides based on the competitive reaction strategy. Reproduced with permission.^[^ ^171^ ^]^ Copyright 2021, Wiley‐VCH.

MoS_2_ as EM wave absorbers suffer from conductivity problems. For MoS_2_, there are two kinds of phases, that is, 2H phase and 1T phase. Ning et al.^[^ ^170^ ^]^ managed the EM wave absorbing mechanism and performance by manipulating phase content toward MoS_2_ through external magnetic field adjustment (**Figure** **10**a). A phase transition from 1T to 2H is found when external applied magnetic field strength increases. During phase transition process, interfacial polarization, defect‐induced polarization, and conductivity loss can be readily regulated and altered EM wave absorbing behavior. Among samples, the one with 50% of 1T phase delivers the best EM wave absorption performance as shown in Figure 10b. Optimized interfacial polarization, defect polarization, and conductivity loss are responsible for excellent EM wave attenuation (Figure 10c).

To solve the above problem for sulfides, our group proposed a competitive reaction mechanism for the preparation of serial metal sulfides to tailor their EM wave absorption behaviors in virtue of varied solubility product constant of metal ions.^[^ ^171^ ^]^ CuS was selected as substrate to investigate competitive reaction due to its high conductivity and highest solubility product constant of Cu^2+^ among Mg^2+^, Mn^2+^, Fe^3+^, Co^2+^, and Ni^2+^ (solubility product constant increased from Mg^2+^ to Ni^2+^). Thanks to this solubility product constant variation, constitution of products could be delicately tuned (Figure 10e). It is found that components, interfaces, and defects can be modulated to adjust interfacial polarization and defect‐induced polarization, thus leading to a wide EAB of 6.8 GHz at middle frequency from 6.3 to 13.1 GHz (Figure 10f). Driven by the competitive reaction, suitable interfacial polarization loss, conductivity loss, and defect‐induced polarization loss jointly lead to superb EM wave dissipation, which is schematic in Figure 10g.

#### 4.1.2 Metal/Metal Oxides

**Figure 11:**
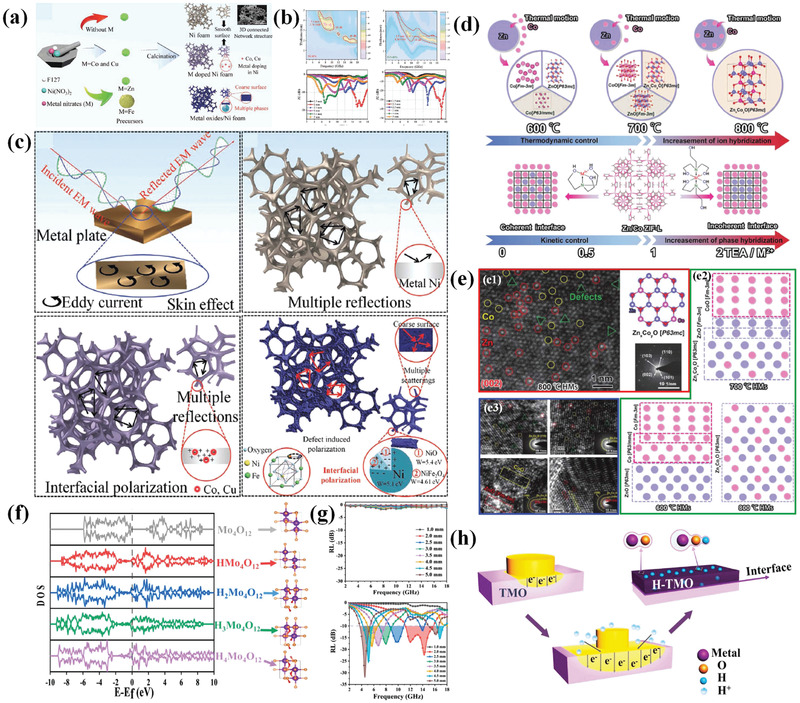
a) Schematic diagram for the synthesis of Ni foam‐based materials, b) RL value curve for bare Ni foam and metal oxide coated Ni foam, c) schematic diagrams of EM wave absorption mechanisms for bulk metal, bare Ni foam, metal‐doped Ni foam, and NiFe_2_O_4_/NiO/Ni foam composites. Reproduced with permission.^[^ ^172^ ^]^ Copyright 2021, Wiley‐VCH. d) Schematic illustration of TEA‐assisted Zn/Co controllable deposition and Zn and Co thermal motion during high‐temperature crystallization, e1) HRTEM images, schematic structure, and SAED images of 800 °C HMs obtained without TEA. e2) Schematic multiphase structure of samples obtained at different temperatures. e3) HRTEM and SAED images of samples at 700 °C with varied TEA addition amount. Reproduced with permission.^[^ ^176^ ^]^ Copyright 2021, Wiley‐VCH. f) Simulated density of states (DOS) of H‐MoO_3_ with different content of hydrogen in the lattice, g) RL value curves before and after hydrogenation of MoO_3_ samples, h) MoO_3_ semiconductor was used as a representative to illustrate the mechanism of proton hydrogenation, and the electron cloud distribution of Mo‐O and Mo‐O‐H. Reproduced with permission.^[^ ^177^ ^]^ Copyright 2021, Elsevier.

Metal materials suffer from high‐density issues and severe impedance mismatch problems when employed as EM wave absorbers. Even though metal nanomaterials or combining metal with others have been reported to overcome above difficulties, new problems such as agglomeration and poor chemical stability of metal nanomaterials due to high surface energy and specific surface appear. In consequence, research on metal‐based EM wave absorbers is scanty. Alternatively, our group provides a strategy for preparing lightweight Ni foam as metal substrate to construct high‐performance metal‐based EM wave absorbers (**Figure** **11**a).^[^ ^172^ ^]^ Trace metal oxides are in situ generated on Ni foam surface to regulate interfaces, defects, and conductivity of final samples. It is found that optimized NiO/NiFe_2_O_4_/Ni foam composite exhibits the best EM wave attenuation capacity due to their strongest interfacial polarization (Figure 11b,c). Though commercial Ni foam has also been reported as substrate to fabricate metal‐based EM wave absorbing materials,^[^ ^173^, ^174^, ^175^ ^]^ the binding between substrate and loading is weak and loading may be not uniform. Besides, rigid feature of commercial Ni foam requires to be ground before EM parameters test, which thoroughly destroys its original morphology characteristic. As such, test results are not accurate. On the contrary, metal oxides obtained in this route are simultaneously formed with Ni foam generation, which ensures the strong binding force and uniform distribution. Moreover, flexible feature of samples renders materials well dispersibility in paraffin without being ground, which can reveal the actual EM wave dissipation of samples. This method provides a new approach for metal‐based EM wave absorbers. In addition, metal foam alloys may also be attained through this route and act as excellent EM wave absorbing materials.

Recently, a thermodynamic and kinetic control strategy is proposed to construct Zn/Co bimetal HMs with tuning ion and phase hybridization for synergistic effect on EM properties (Figure 11d).^[^ ^176^ ^]^ Kinetic control process is achieved by different combination ability of Zn and Co ions toward chelating agent triethanolamine (TEA) while thermodynamic control is realized through gradient temperature treatment. At a low calcination temperature of 600 ℃, interfacial polarization is the dominated loss mechanism due to the coexistence of hexagonal ZnO, cubic Co, and transition state hexagonal Co when TEA content increment from 0 to 8 mmol. While for calcination temperature at 800 ℃, Co enters into lattice of ZnO thus defect induced polarization is the dominated loss mechanism. TEA amount ascent results in a decline in defect sites and subsequent polarization process. The defects and schematic phase transition are shown in Figure 11e. Under suitable experimental conditions, interfacial polarization and defect‐induced polarization can be simultaneously tailored to achieve best EM wave loss behavior.

Low electrical conductivity of semiconductor metal oxides restricts their application as high‐performance EM wave absorbers. In allusion to this problem, Cheng et al.^[^ ^177^ ^]^ proposed a proton hydrogenation strategy to delocalize electrons in metal oxides, thus promoting their conductivity and EM wave attenuation. In detail, proton hydrogenation process is accomplished by dissolution of commercial metal oxides into Zn/HCl reaction system. Hydrogenation degree that affects EM wave loss is tuned through different reaction times. On basis of simulation results in Figure 11f, prolonged reaction will lead to movement of conduction band towards lower energy region and subsequent higher conductivity. After this treatment, original MoO_3_ sample with no EM loss capacity changes into effective absorber according to RL values in Figure 11g. The mechanism of proton hydrogenation and the electron cloud distribution of Mo—O and Mo—O—H is shown in Figure 11h. This strategy is effective to other transition metal oxides including Nb_2_O_5_ and WO_3_.

### 4.2 External Condition Stimulation

**Figure 12:**
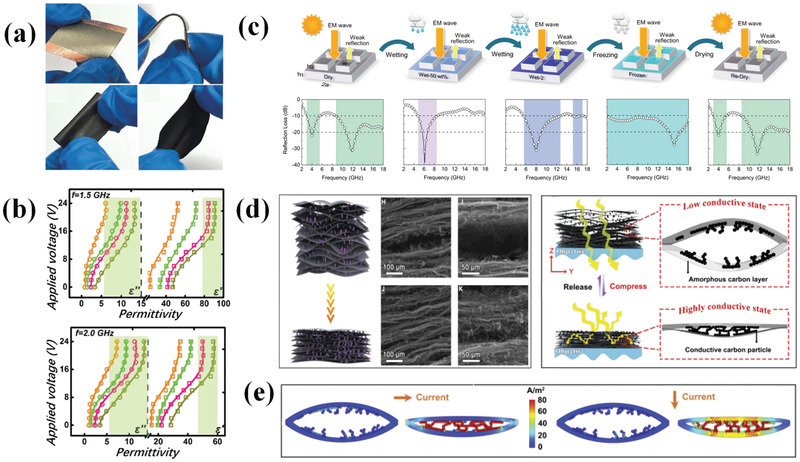
a,b) Digital photographs of SnS/SnO_2_@C flexible device and interlayer absorption film, dielectric parameters of the flexible device with applied voltage. Reproduced with permission.^[^ ^178^ ^]^ Copyright 2018, Wiley‐VCH. c) Experimental weather‐manipulated absorption performance of metamaterials by the arch method: illumination of structure (top) and measured absorption performance (bottom) at various conditions. Reproduced with permission.^[^ ^182^ ^]^ Copyright 2018, American Chemical Society. d) Schematic illustration of microstructure evolution of the WXC aerogel during compression and corresponding SEM images and schematic illustration of the off/on shielding performance of the switchable EMI, e) Simulation of current density in 2D structure. The arrows indicate the current directions (from left to right and from top to bottom). Reproduced with permission.^[^ ^183^ ^]^ Copyright 2021, Elsevier.

Despite composition and morphology manipulation, the EM parameters of absorbing materials can also be regulated by changing external conditions. For instance, Lv et al.^[^ ^178^ ^]^ utilized a voltage‐boosting strategy to realize the absorption of EM wave in low frequency by tuning conductivity and subsequent EM parameters. They selected core‐shell SnS/SnO_2_@C composite as representative and constructed them into flexible devices (**Figure** **12**a) to clarify the influence of external voltage on the improvement of EM wave attenuation at a frequency range of 1.0–2.0 GHz. First, they obtained suitable real part *ε*′ and imaginary part *ε*″ values scope for well EM wave absorption in low frequency through calculation and detailed value ranges are *ε*′ = 80–100 and *ε*″ = 5–40 at 1.5 GHz while *ε*′ = 45–60 and *ε*″ = 5–30 at 2 GHz. The bare composite show undesirable parameters but the values gradually increase and locate at suitable position along with applied bias voltage increased from 0 to 24 V (Figure 12b), thus exhibiting excellent EM wave absorption performance from 1.5 to 2 GHz. Except for this work, electric stimulation strategy is also reported in Sn/SnS/SnO_2_@C core‐shell composite, Fe@C composite, and graphene electrodes^[^ ^179^, ^180^, ^181^ ^]^ materials to adjust their EM characteristics.

Except for this inspiring strategy, there are also works that are worth learning. Zhang et al.^[^ ^182^ ^]^ attempted to regulate EM wave absorption performance of sample by taking advantage of polar water and ice in environment. In simulative conditions, a frequency selective function can be realized at different weathers (Figure 12c). The change in EM wave loss is ascribed to dipolar polarization and interfacial polarization enhancement brought about by polar water and ice. Liu et al.^[^ ^183^ ^]^ reported a switchable smart EM shielding aerogel through compression and decompression. The SEM images and corresponding states are shown in Figure 12d. On basis of their explanation of EM shielding and transparent transition, original aerogel shows well‐matched impedance due to the existence of abundant air and possesses low conductivity, thus behaving EM transparent feature. After compression process, air is eliminated and a conductive network is formed, thus leading to mismatched impedance and subsequent EM shielding. The strengthened current density from stimulation results in Figure 12e substantiates the above assumption. During this process, an intermediate state that both impedance match and suitable conductivity may be achieved to reach excellent EM wave absorption performance. Overall, it is a promising pathway to take advantage of external conditions such as electric field, magnetic field, thermal energy, pressure, and other conditions to regulate EM loss mechanisms to realize desirable wave absorbing behaviors.

Other than the above strategies, it has also been reported that EM wave loss mechanism can be tuned by modulating incident angles. Notably, oblique incidence is widely investigated in metamaterial EM wave absorbers due to their unique patterns. It is also evident that this route can also be utilized to manipulate EM wave absorption behaviors and underlying mechanisms for conventional absorbing materials.^[^ ^184^, ^185^, ^186^, ^187^, ^188^, ^189^, ^190^, ^191^, ^192^ ^]^ One effect of oblique incidence is that impedance matching characteristics can be regulated under different incident angles.^[^ ^187^, ^188^ ^]^ Besides, transverse electric (TE) mode and transverse magnetic (TM) mode would affect EM wave propagation path. It is found that absorbers can no longer obtain effective absorption for TE mode but another efficient absorption region could be observed at higher frequencies even when the incident angle exceeded 80° for the TM case. To Figure out this phenomenon, Zhang et al.^[^ ^192^ ^]^ took advantage of carbonyl iron and polyurethane composite as EM wave absorber and discovered that changes in the reflected waves at different interfaces were responsible for the varied behaviors. For TE mode, increment of incident angles would broaden the values’ difference between wave reflected directly from the top interface (R1) and high‐ordered reflections wave (R2). In other words, more EM wave is reflected and less EM energy is consumed by absorber. Eventually, EM wave absorption decline at a higher angle is observed for TE mode. TM mode, however, exhibits different mechanisms. EM wave absorbers with a suitable thickness could achieve efficient absorption at high incident angles due to the combined effect of interference cancellation and intrinsic loss. Overall, EM wave absorption mechanisms are not only based on absorbing materials but also referred to multiple reflection models. Despite their inspiring achievement, researches on oblique incidence for conventional EM wave absorbing materials are scarce and more attention is needed to get further development.

## 5 Conclusion and Outlook

In conclusion, dielectric loss mechanism investigation is of great significance to understand EM wave attenuation behaviors and to guide the design of novel absorbing materials. In this review, we provide a comprehensive view toward dielectric loss mechanisms including interfacial polarization, dipolar polarization, conductive loss, and defect‐induced polarization. Some concepts and corresponding data support for each mechanism are introduced. Particularly, some misunderstandings and ambiguous concepts for each mechanism are highlighted. Especially for the defect‐induced polarization, the presence of different defect sites including point defect (vacancy and heteroatoms doping) and other defect sites (line defect and planar defect) may lead to different induced polarization process such as dipolar polarization and space charge polarization. Accordingly, defect‐induced polarization is individually concluded to describe their contribution to EM wave loss. Besides, typical researches on the main EM wave loss mechanism shed light on the innovation of delicate materials design to realize main loss mechanism determination and novel dielectric loss mechanisms are emphasized. Moreover, new dielectric loss mechanism regulation strategies including novel synthetic routes for metal sulfides and metal/metal oxides as well as external condition stimulation instead of regular components compositing are summarized to provide inspiring thoughts toward simple and effective EM wave attenuation behavior modulation. To have a comprehensive comparison, some typical EM wave absorbing materials, their loss mechanisms, and detailed absorption parameters are summarized in **Table** **1**. It is not hard to find that interfacial polarization can be brought about in absorbing composite. Just because of the introduction of multiple components, subsequent interfaces, defect, and conductivity are also changed. Therefore, it is still formidable task to Figure out the contribution degree of each mechanism.

**Table 1:** Summarized EM wave loss mechanisms and absorption performance of some typical absorbing materials

Absorber	Dominant loss mechanism	Absorber Content [%]	Thickness [mm]	EAB [GHz]	RL_min_ [dB]	Ref.
TiO_2_@Fe_3_O_4_@PPy	Interfacial polarization and conductive loss	40	2.2	6	−61.8	[83]
Fe/MFe_2_O_4_	Interfacial polarization	50	1.5	6.2	−27	[85]
Ti_3_C_2_T_x_ Foam	Interfacial polarization, conductive loss and dipolar polarization	3.3	1.4	4.2	−27.2	[88]
Multi‐shell carbon spheres	Interfacial polarization and conductive loss	25	2.8	4.2	−48.5	[91]
SiC_nw_@SiC Foam	Interfacial polarization and conductive loss	‐	2.82	5.6	−52.5	[93]
Multi‐shelled MCo_2_O_4_	Defect induced polarization	50	1.86	5.84	−48.1	[119]
rGO	Defect induced polarization and conductive loss	30	3.5	5.92	−37.2	[121]
ZnO/ZnS/CuS	Interfacial polarization, conductive loss and dipolar polarization	33	1.59	5.2	−43.9	[123]
NS‐rGO	Defect induced polarization and conductive loss	2	3.6	6	−51.9	[128]
VO_2_	Defect induced polarization and conductive loss	‐	1.5	5.2	−52.4	[131]
Hydrogenated TiO_2_	Interfacial polarization	60	‐	‐	‐	[138]
Ni@C@ZnO	Interfacial polarization and conductive loss	25	2.5	4.1	−55.8	[140]
TiC@C	Interfacial polarization	50	2.1	5.3	−45.1	[144]
MOF‐74‐S1	Interfacial polarization	40	2.3	7.6	−33.5	[146]
Co_1.29_Ni_1.71_O_4_ hollow sphere	Defect induced polarization	50	2	5.13	−44.5	[149]
2H/1T MoS_2_	Interfacial polarization, conductive loss and dipolar polarization	50	3.5	3.89	−45.5	[170]
Cu‐Co sulfide	Interfacial polarization and defect induced polarization	33	2.8	6.8	−45.7	[171]
Ni foam/NiO /NiFe_2_O_4_	Interfacial polarization	10	2.5	8.8	−50.0	[172]
Zn/Co HMs	Interfacial polarization and defect induced polarization	33	1.6	4.8	−45.9	[176]
H‐MoO_3_	Interfacial polarization and conductive loss	70	1.9	4.64	−33.7	[177]

Though tremendous advances have been achieved in EM wave absorption field, there are still many research aspects that should be taken into consideration.

a) There are still many rooms for EM wave loss mechanisms investigation and regulation. For defect‐induced polarization investigation, more attention has been paid to the research of anion vacancies (such as oxygen vacancy and sulfur vacancy), while a study on cation vacancies is rarely reported. Despite of their high formation energy, generation of cation vacancies is also reported.^[^ ^193^ ^]^ Thus, cation vacancies and their effects to EM wave loss could be illuminated. For interface engineering, crystal‐plane engineering can be considered as referred above. Moreover, interfacial effect can also be regulated by designing 2D/2D materials and changing their contact area or the bonding form modulation such as chemical bond binding, van der Weals forces to investigate their influence on EM wave dissipation. In addition, EM wave loss mechanism manipulation through external stimulation such as thermal heat, EM field, and other conditions hold great promising to accomplish EM wave attenuation behaviors adjustment even in untamed low working frequency.
b) With respect to novel models for EM wave absorbers, there are also some alternatives. For instance, it has been reported previously that Fe_3_O_4_ as core in core‐shell is moveable under independent electronic field or magnetic field.^[^ ^194^ ^]^ As such, moveable core in core‐shell structure design can be regarded as magnetic dipole or electronic dipole and will consume EM energy by polarization and relaxation under external alternating EM field. It has to be pointed out that the moveable core is realized in water media in literature. Therefore, their application may be suitable underwater or combined with the water‐based metamaterials.^[^ ^195^ ^]^ Besides, advantage of multi‐shelled structure can be utilized to investigate interfacial polarization effect. For example, we can prepare shell composition identical materials but change their arrangement in different order. Multi‐shelled metal oxides with A@B@C structure, where A, B, and C are different oxides. By modulation their arrangement order, we can manipulate their interfacial effect and subsequent interfacial polarization process, thus investigating the relation between interfacial polarization and EM wave absorption performance.
c) The multi‐functional absorbing materials have become an urgent need for next‐generation EM wave absorbers. Apart from lightweight, thin thickness, wide EAB, and strong absorbing capacity, other functions including flame‐retardant, thermal stealth, water‐proof and self‐cleaning, oxidation and corrosion resistant, flexible and wearable, near‐infrared absorption, self‐healing, and optically transparent characteristics are also required for their practical applications.^[^ ^196^, ^197^, ^198^, ^199^, ^200^, ^201^, ^202^, ^203^, ^204^, ^205^, ^206^ ^]^ Currently, a solution to realize different functions is utilizing specific components. For instance, flame‐retardant and thermal stealth functions can be accomplished by utilizing polyurethane or carbon (including graphene and biochar) aerogel. While for water‐proof and flexible features, the newly emerging MXene material is a suitable alternative. MXene owns numerous advantages when acting as EM wave absorbing materials. As a high conductive material, it can provide strong conductive loss. Meanwhile, Mxene family with abundant members could be fabricated through different reaction conditions, which endows them controllable defects and components and subsequent polarization loss ability. Furthermore, diverse polar functional groups on Mxene also give rise to excellent dipolar polarization loss. In view of its macroscopic scale assemblies, flexible features, corrosion resistance characteristics, Mxene based materials can also serve as multifunctional EM wave absorbing materials. Though compositive EM wave absorbers could be formed by compositing different parts, it is undoubtedly that this process contains complex protocols and is not beneficial to scalable products. Find a scalable and cost‐effective access to multi‐functional EM wave absorbers and realize their practical application is highly desired. Therefore, more efforts should be devoted to this research aspect.
d) Heat energy is generated during the propagation of EM wave in absorbers. How to take advantage of this energy or utilize the temperature‐rising characteristic of absorbing materials to be applied in related fields is also attractive. As mentioned previously, Cao's group used the heating energy during EM wave absorption and well thermal conductivity of graphene to elevate temperature of absorbers to achieve higher conduction loss. In their further study, a high conductive material delaminated titanium carbide MXene nanosheet was selected as EM wave absorber to construct thermoelectric generator.^[^ ^207^ ^]^ The thermoelectric generator contains P‐Type and N‐type semiconductors that are connected by high thermal‐conductivity and non‐conductive aluminum alloy, whose surface is coated with 80% of absorbing materials. The heat energy generated by conduction loss will induce temperature difference current between two semiconductors and supply power for low‐power electric devices. Lv et al.^[^ ^208^ ^]^ also put up with a strategy to transfer heat energy into electric energy. They take advantage of Sn@C composite and its biological cell‐like splitting behavior under calcination treatment to realize both of high thermoelectric Figure of merit of 0.62 at 473 K and EM wave absorbing capacity. Apart from energy usage, Zhu et al.^[^ ^209^ ^]^ found that temperature‐rising characteristic of materials under EM field was beneficial to promote catalytic performance. With the aid of microwave irradiation, catalysts with EM wave absorbing capacity would increase the temperature of reaction system, which is thermodynamically favorable for higher catalytic reactivity. As such, effort on investigation of novel energy transition and interdisciplinary applications should also be devoted.

Noticeably, the development of advanced characterization techniques such as in situ application of microwave fields in TEM is of great significance to further EM wave loss mechanism study but remains an enormous challenge. Nevertheless, we are confident that dielectric loss mechanism investigation will receive tremendous reward and be helpful to accomplish novel EM wave absorbers design.

## Conflict of Interest

The authors declare no conflict of interest.
